# Density functional theory investigation of mechanisms of degradation reactions of sulfonated PEEK membranes with OH radicals in fuel cells: Addition-elimination reactions and acid catalyzed water elimination

**DOI:** 10.21203/rs.3.rs-2565467/v1

**Published:** 2023-02-10

**Authors:** Jonathan E. Stevens, Courtney M. Pefley, Alice Piatkowski, Zachary R. Smith, Nikolina Ognanovich

**Affiliations:** University of Detroit Mercy; University of Detroit Mercy; University of Detroit Mercy; American Axle Manufacturing; University of Michigan

**Keywords:** density functional theory, M062X, fuel cell membrane, sPEEK, hydroxy radical

## Abstract

Sulfonated polyether (ether) ketone, or sulfonated PEEK (sPEEK) membranes are one possible candidate for proton-transfer membranes in hydrogen fuel cells. Reaction with hydroxy radicals is expected to be a significant source of degradation of these membranes during fuel cell operation. In this work, the reactivity of the sPEEK polymer molecule with OH radicals is studied by M062X hybrid density functional calculations of the energetics of several reaction paths in a water environment as modeled by polarized continuum model (PCM) calculations. Reactants, products, encounter minima and transition states are optimized for a reaction pathway in which OH addition is followed by acid-catalyzed water elimination which cationizes the polymer, degradation is expected to follow this reaction as the unstable cation then undergoes bond-breaking or other reactions. Two pathways for this acid-catalyzed cationization, one in which a water molecule plays the role of an additional co-catalyst, are reported. Further calculations explore reaction pathways in which addition of OH to the polymer is followed by bond breaking reactions which would break the polymer chain or the bond between the polymer and sulfonyl groups. Examination of the free energy barriers to all these reactions, relative to reactants, suggest that these direct bond-breaking reactions may compete somewhat with acid-catalyzed water elimination following OH addition.

## 1. Introduction

Fuel cells of particular interest for automotive applications are proton-exchange membrane fuel cells (PEMFCs)[1,2]. A key component of such devices is the proton exchange membrane (PEM)[1], a semipermeable membrane which allows protons produced at the anode to migrate through to the cathode of the fuel cells[2]. Currently, proton exchange membranes are typically perfluoroalkylsulfonic acid (PFSA) membranes[1,2], especially Nafion[2–5]. However, fluorinated membranes and fluorinated polymers in general present environmental hazards upon degradation[6,7,8].

**Figure 1:**
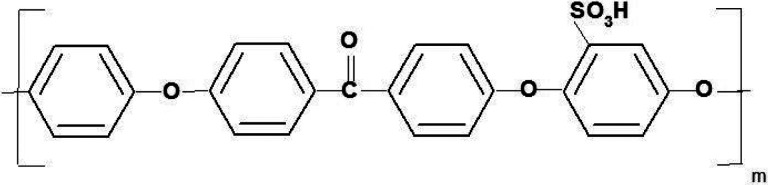
Repeating unit of SPEEK polymer

One of several proposed non-fluorinated alternatives to PFSA membranes is sulfonated aromatic membranes, such as the polymer sulfonated polyether(ether) ketone, or sulfonated PEEK (sPEEK)[2,6,7], displayed in Figure 1.

Both PFSA and sulfonated aromatic membranes such as sPEEK are expected to be subject to degradation during fuel cell operation through reaction with OH[1,7,9,10], H[1,6,9,11], and OOH[1,9,11] radicals or H_2_O_2_[12,13] molecules. In the case of Nafion this degradation has been studied experimentally [9,12,14,15] and there have also been recent computational works on the degradation reactions of Nafion with OH and H radicals[5,16,17] with H_2_O_2_[12], and most recently with a hypothesized H_3_O radical[18].

In the case of sPEEK, degradation has been studied experimentally with electron spin resonance methods[10,11,19]. Huber and Roduner[10] studied the reactions of a number of sulfonated aromatic model molecules with OH radicals. EPR experiments observe the formation of benzyl radicals in aqueous solution, and suggest this phenomenon initiates from an acid catalyzed loss of water from hydroxycyclohexadienyl radicals formed by the attachment of OH to the aromatic ring. This proposed mechanism is consistent with a mechanism reported for the production of benzyl radicals in reactions of toluene[20] and other methylbenzenes[21]. In the case of sPEEK, this process is expected to occur through a process of OH addition to the aromatic ring, followed by protonation of the OH adduct, then elimination of water[1,10,20,21]. This reaction would this cationize the membrane[1], leading to chain-breaking, cross-linking, or further hydroxylation reactions.

More recent experiments have used spin-trapping methods to explore the reactions of sPEEK with OH directly both in the *in situ* environment of an operating sPEEK-membrane hydrogen fuel cell[11] and in *ex situ*[19] experiments. *Ex situ* experiments found evidence of membrane degradation, detecting both phenoxy and phenyl radical products from sPEEK exposure to OH. In situ experiments observed no membrane degradation products, although the authors suggest such radicals would appear if longer fuel cell run times had been used.

Computational investigation of the degradation reactions of sPEEK has been reported in two works. Panchenko[7] has examined the thermodynamic feasibility of some reactions of non-fluorinated sulfonated aromatic membranes with OH radicals, focusing on model molecules for the polymers sPEEK and PSU (polyethersulfone). The calculations studied the thermodynamics of two types of reactions: abstraction of H atoms in sPEEK and PSU by OH groups, and also on the attachment of OH groups to some of the carbon atoms on the aromatic rings present, followed by reactions with O_2_ molecules.

This work implemented the B3LYP method with 6–31G(d) and 6–311+G(d, p) basis sets and used the polarizable continuum model (PCM) to incorporate effects of solvation in water. In some cases, sulfonic acid groups were modeled in both protonated and deprotonated form.

**Figure 2:**
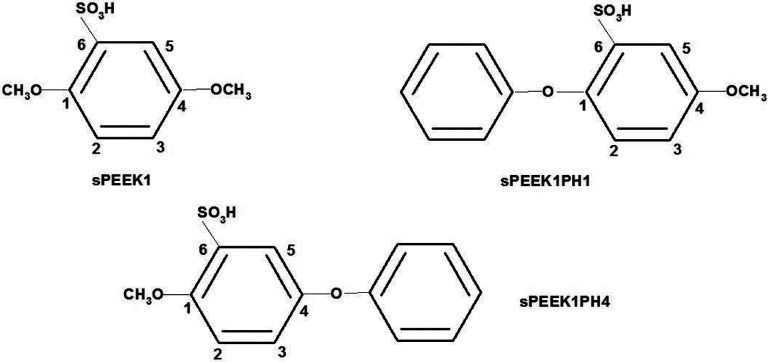
Model molecules for sPEEK

The second work[6] studied the reactions of sPEEK with H radicals in a solvated environment with M062X/6–311+G(2d,2p) optimizations, followed by M062X/6–311+G(3df,2p) singlepoint calculations performed on optimized structures. To represent sPEEK, this work employed the model molecule sPEEK1, one of the model molecules originally implemented in reference 7. This molecule is displayed in Figure 2. As seen in the figure, the carbon atoms of the aromatic ring are numbered 1–6. These carbon atoms are sites for radical attachment as well as proton attachment, as seen in reference 6 and in this work.

This study found that addition of H radicals to carbon atoms at sites 1 and 4 of SPEEK1 produces adduct structures in which the C-OCH_3_ linkage, the portion of the model corresponding to the ether bridge in the sPEEK polymer, becomes extremely fragile; in fact, breaking of these bonds following addition are found to be slightly exergonic processes. Transition state optimizations found that these bond breaking reactions have low free energy barriers; only 4–8 kcal/mol relative to the adducts, and below the relative free energy of the sPEEK1 + H reactants. Similarly, the addition of H radical to the carbon at site 6 produces an adduct structure in which C-S bond breaking is slightly exergonic and in which the barrier to bond breaking is small (~ 7 kcal/mol).

The addition-elimination reactions of sPEEK1 with H suggest similar reactions might occur upon OH addition to sites 1–6 in the aromatic ring of that molecule. In the case of addition at sites 1 and 4, the expected result is phenoxy radicals such as those detected in the *ex situ* spin trap experiments[19]. Addition of OH radicals at site 6 might also be expected to produce HSO_3_ radicals in the same fashion as addition of H radicals. It may be speculated that such reactions may compete with the acid-catalyzed elimination reaction in the phenoxy radicals.

This work provides the first computational density-functional determination of the mechanism of the acid-catalyzed water elimination reaction resulting from exposure of sPEEK to OH radicals in an aqueous environment. In addition, addition-elimination reactions corresponding to C-O chain breaking and C-S bond breaking reactions are also calculated. These computations employ solvated density functional M062X/6–311+G(2d,2p) calculations of the potential energy surface of the reactions of sPEEK model molecules with OH radicals and H_3_O^+^, with singlepoint calculations with a larger basis set at all optimized geometries, to provide improved energetics. The chosen methods provide complete consistency with the calculations implemented within reference 6.

In this manuscript, all species optimized with these solvated density functional M062X/6–311+G(2d,2p) calculations will be named in bold. Cartesian coordinates for all these structures are provided, in the order mentioned in this article, in the Supplementary Information for this article (see Online Resource 1).

The model molecules include **SPEEK1** as noted above, as well as additional molecules **SPEEK1PH1** and **SPEEK1PH4** also displayed in Figure 2. The latter two replace −OCH_3_ moieties at sites 1 and 4 with phenyl rings. Model molecules **SPEEK1PH1** and **SPEEK1PH4** provide more realistic polymer models and hence improved thermodynamics and barriers for the computations of C-O bond breaking reactions at sites 1 and 4. The following, second section of this work describes the methods of these calculations in detail. The third section, Results, describes the formation of sPEEK hydroxyl radicals and discusses all the optimized reaction pathways in detail, displaying energetics with respect to OH-sPEEK adduct and protonated OH-sPEEK adduct molecules. The fourth section, Discussion and Conclusion, then summarizes the results in terms of reactions on the sPEEK + OH + H_3_O^+^ potential energy surface, with one of the models **SPEEK1**, **SPEEK1PH1** and **SPEEK1PH4** assuming the role of sPEEK in calculation of the energetics.

## 2. Computational Methods

The Gaussian 16[22] electronic structure packages were used for all calculations. Unrestricted density functional M062X[23], calculations with the 6–311+G(2d,2p) basis set (M062X/6–311+G(2d,2p)) optimized all reactants, products, intermediates, and transition states, and determined reaction energetics. For the initial M062X/6–311+G(2d,2p) calculations, frequency calculations characterized each structure as a minimum or transition state, and provided enthalpy and free energy corrections to the base energy. The connectivity of all transition states to reactants and products was determined by Intrinsic Reaction Coordinate (IRC) calculations.

Single-point M062X/6–311+G(3df,2p) calculations at optimized geometries provided improved energetics. For M062X/6–311+G(3df,2p)//M062X/6–311+G(2d,2p) calculations, the enthalpy and free energy corrections are taken as those found by M062X/6–311+G(2d,2p) frequency calculations. For all calculations, solvent (water) modeling is provided by the integral equation formalism polarized continuum model[24] (IEFPCM or PCM) as implemented in the Gaussian program. The free Avogadro[25] program was used to visualize optimized structures and also visualize computed imaginary vibrational modes in transition state structures.

The potential energy surface thus generated is thus compatible with recent work on the model sPEEK1 molecule with hydrogen atoms[6].

## 3. Results

### 3.1 Addition of OH to model molecule SPEEK1

The solvated M062X/6–311+G(d,p) optimizations show that the addition of OH radical (**OH**) to sites 1–6 of model molecule **SPEEK1** is thermodynamically spontaneous and occurs without reverse barrier. Table 1 shows the enthalpies and free energies of formation of adducts at each of the six sites. While enthalpies and free energies of addition of H radicals to **SPEEK1** were shown to diverge by up to ~8.4 kcal/mol[6], the enthalpies and free energies for the formation of OH adducts are more similar, varying by only 2.4 kcal/mol in the case of the enthalpy and 1.4 kcal/mol in the case of the free energy. Table 1 also shows the enthalpy and entropy of addition provided by solvated B3LYP/6–311+G(d) calculations as shown in reference 7. In general, the computations of this work predict more exothermic and exergonic additions than reference 7.

### 3.2. Elimination following addition of OH at site 1

**Figure 3:**
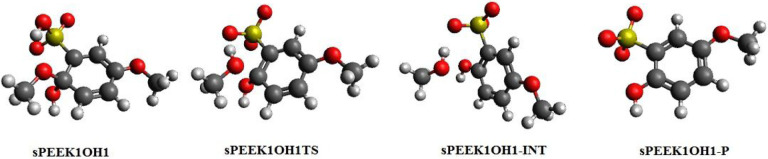
Optimized structures for elimination reaction following addition of OH to the carbon atom at site 1 of sPEEK1

The **SPEEK1OH1** adduct forms at site 1 with enthalpies and free energies relative to **SPEEK1** + **OH** as found in Table 1. A C-O bond breaking transition state **SPEEK1OH1TS** appears at an small enthalpy and free energy relative to the adduct, and connects the adduct to intermediate **SPEEK1OH1**-**INT**, at lower enthalpy and free energy to the adduct. This structure is a hydrogen-bonded complex of methanol to a molecule in which −OH replaces −OCH_3_ at site 1 and the H has been abstracted from the sulfonyl group at site 6, leaving an unpaired electron. The overall reaction leading to the separated products is slightly exothermic (−3.4 kcal/mol) and exergonic (−15.1 kcal/mol), as summarized in Table 2. Figure 3 displays optimized structures on the reaction pathway.

The optimized structure for this radical is named **SPEEK1OH1**-**P**. The final products, methanol (**CH3OH**) and **SPEEK1OH1**-**P** are found without reverse barrier to the intermediate and are at an enthalpy of −2.9 (−3.4) relative to the adduct. Figure 3 displays optimized structures on the pathway of this reaction. The elimination/hydrogen abstraction process reported here is also shown to occur at site 1 following addition of H radicals at site 1[6]; namely, the elimination process in that reaction also produces methanol and a species with an unpaired electron on the −SO_3_ group.

M062X/6–311+G(2d,2p) calculations also computed the addition of OH to site 1 of **SPEEK1PH1** to form **SPEEK1PH1OH1**, and determined a transition state **SPEEK1PH1OH1TS** for breaking of the C-O bond at site 1. As is also shown in Table 2, replacement of a methyl group for a phenyl group at site 1 of the model molecule produces a transition state with an enthalpy of only 3.7 kcal/mol relative to the reactant, which is 1.0 kcal/mol lower than in the case of the methyl substituent. The free energy of the transition state is also lowered by phenyl replacement of the methyl group, 4.7 kcal/mol as opposed to 5.6 kcal/mol. Phenyl replacement at site 1 might be anticipated to produce a similar lowering of barriers by ~1.0 kcal/mol to reaction for the bond-breaking reaction following addition of H radical at site 1 of **SPEEK1** as described in reference 6.

IRC calculations for this transition state confirm the connectivity of **SPEEK1PH1OH1TS** to **SPEEK1PH1OH1** in one direction of the reaction; in the other direction, IRC calculations connect the transition state to **SPEEK1PH1OH1**-**INT**. This structure is a hydrogen-bonded complex of separated products phenol (**PHENOL**) and **SPEEK1OH1**-**P**.

Table 2 summarizes the binding enthalpy and free energy of the OH adduct to **SPEEK1** and **SPEEK1PH1**, as well as the relative enthalpy and free energy of the transition state and products.

### 3.3. Elimination following addition of OH at site 4

Table 1 shows the enthalpy and free energy of formation of the **SPEEK1OH4** adduct from **SPEEK1** + **OH**. Energetics of structures for a C-O bond breaking reaction are shown in Table 3. A C-O bond breaking transition state **SPEEK1OH4TS** appears at an enthalpy of 19.3 kcal/mol and free energy of 18.9 kcal/mol relative to the adduct. This transition state lies at a much higher enthalpy or free energy relative to the adduct than is the case for bond breaking at site 1.

IRC calculations connect **SPEEK1OH4TS** to **SPEEK1OH4** in one direction and to **SPEEK1OH4**-**INT** in the other, the latter is a intermediate complex in which an OCH_3_ radical is hydrogen-bonded to the sulfonyl hydrogen on the phenolic product molecule. Intermediate **SPEEK1OH4**-**INT** lies at an enthalpy of ~5.0 kcal/mol relative to the adduct. As indicated by the structure of the intermediate exit complex, no hydrogen abstraction from the sulfonyl group takes place during the addition-elimination reaction at site 4, and the final products include a CH_3_O radical (**CH3O**)and a phenolic molecule in which OH has replaced OCH_3_ substituent at site 4 (**SPEEK1OH4**-**P**) The final separated products **SPEEK1OH4**-**P** + methoxy (**CH3O**) are found at an enthalpy of 9.7 relative to the adduct; G is −2.1 relative to the adduct, for a slightly exergonic reaction.

M062X/6–311+G(2d,2p) calculations also computed the addition of OH to site 1 of **SPEEK1PH4** to form **SPEEK1PH4OH4**, and determined a transition state **SPEEK1PH4OH4TS** for breaking of the C-O bond at site 4. IRC calculations were carried out to establish the connectivity of this transition state to **SPEEK1PH4OH4** in one direction and to **sPEEK1PH4OH4**-**INT**, a hydrogen bonded complex of the final products, **PHENOXY** and **SPEEK4OH4**-**P**.

**Figure 4:**
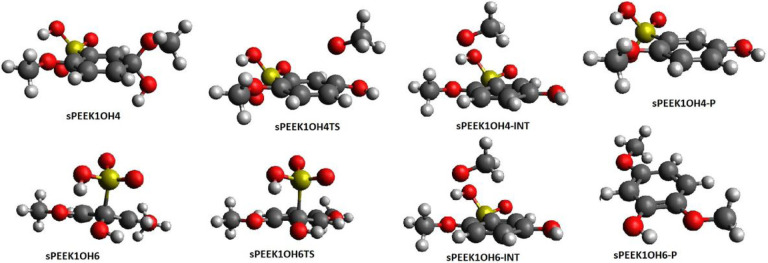
Optimized structures for reactions following OH addition to **sPEEK1** at site 4 (top) and site 6(bottom)

Table 3 summarizes the binding enthalpy and free energy of the OH adduct to site 4 of **SPEEK1** and **SPEEK1PH4**, the relative enthalpy and free energy of the transition state and products. Figure 4 displays optimized structures on the reaction pathway.

Table 3 shows that substitution of phenyl for the OCH_3_ moiety at site 4 lowers the enthalpy and free energy barriers represented by the transition state by ~10 kcal/mol. Phenyl replacement at site 4 might be anticipated to produce a similar lowering of barriers by ~10 kcal/mol to reaction for the bond-breaking reaction following addition of H radical at site 4 of **SPEEK1** as described in reference 6.

### 3.4. Elimination following addition of OH at site 6

#### 3.4.1. Elimination of the HSO_3_ radical

Table 1 shows that the formation of the **SPEEK1OH6** adduct from **SPEEK1** + **OH** is exothermic and exergonic. The enthalpy and free energy of the bond breaking transition state **SPEEK1OH6**TS are displayed in Table 4. IRC calculations connect this transition state to **SPEEK1OH6** in one direction and to a hydrogen-bonded intermediate **SPEEK1OH6**-**INT** in the other. This intermediate is as an HSO_3_ radical hydrogen bonded to the OH group on the phenolic product molecule. The separated products are the HSO_3_ radical **HSO3** and the phenol product **SPEEK1OH6**-P. The C-S bond breaking reaction is slightly exothermic and occurs spontaneously. The barrier to this reaction is very small relative to **SPEEK1OH6**, as noted in Table 4. Figure 4 displays optimized structures on the reaction pathway.

#### 3.4.2. Thermodynamics of other elimination reactions following OH addition at site 6

Reference 6 suggests that in the case of OH radical addition at site 6, H_2_SO_4_ (**H2SO4**) elimination might occur; the other product would be the sPEEK molecule in which the C-S bond at site 6 is broken, leaving an unpaired electron at C6; this molecule is referred to as **SPEEK1OH6**-**P2**. Additional reactions following OH addition at site 6 might involve the production of HSO_4_ (HSO4) producing a sPEEK1 molecule in which −SO3H is replaced by −H, referred to as **SPEEK1OH6**-**P3**. Another possibility would be elimination of H2SO_3_ (**H2SO3**), the other product being a sPEEK1 molecule in which a phenoxy radical replaces −SO3H at site 6, referred to as **SPEEK1OH6**-**P4**. Table 5 summarizes the enthalpy and free energy of the reactions discussed above.

### 3.5. Protonation and water elimination following OH addition at site 3.

#### 3.5.1. Structure and thermodynamics of protonated sPEEK1OH3 complexes; partial charge of likely protonation sites for sPEEK-OH adducts

Site 3 of **SPEEK1** is chosen for computational investigation of the water elimination oxidative process suggested for the degradation of sPEEK membranes[1,10]. Table 1 notes that the **SPEEK1OH3** moiety forms spontaneously and without barrier. The M062X/6–311+G(2d,2p) geometry optimizations do not locate a minimum corresponding to addition of a proton to the hydroxyl oxygen attached to site 3. The nearest sites for protonation to the hydroxyl group are the carbons at site 2 (**SPEEK1OH3H2**+) and site 4 (**SPEEK1OH3H4**+). Protonation at these sites (corresponding to transfer of a proton from an optimized solvated hydronium, **H3O**+, to form the protonated moiety and an optimized solvated water molecule (**H2O**) is both exothermic and exergonic in the case of site 3, as shown in table 6. In contrast, addition to site 4 is slightly endothermic and endergonic, as also shown in table 6.

The difference in the free energy of protonation may be a function of the partial Mulliken charge on the aromatic carbons adjacent to the site of attachment of the OH adduct. The optimized M062X/6–311+G(2d,2p) **SPEEK1OH3** structure exhibits a Mulliken charge of −0.16453 on site number 2 and a Mulliken charge of +0.316640 on site 5. The exergonic addition of proton at site 2 may be a function of the partial negative charge at site 2 to form **SPEEK1OH3H2**+, while the partial positive charge at site 4 may correspond to the endergonicity of protonation at that site.

Table 7 summarizes the partial charges on adjacent aromatic carbons for adducts **sPEEK1OH1** through **SPEEK1OH6**. All adducts other than **sPEEK1OH2** exhibit a negative Mullken charge on at least one aromatic carbon adjacent to the attachment site of the OH radical. An additional solvated M062X/6–311+G(2d,2p) calculation of the OH adduct of benzene, **BZ**-**OH**, provides a partial negative charge of −0.101056 on sites 2 and 6, the sites adjacent to the attachment site (referred to as site 1).

This work presents acid-catalyzed water elimination reactions occurring following protonation of **sPEEK1OH3** at site 2.

#### 3.5.2. Barrier to protonation of sPEEK1OH3

The mechanism of the acid catalyzed elimination reaction studied in this work requires that the **SPEEK1OH3** hydroxylated aromatic ring is protonated by a hydronium molecule. The protonation of aromatic rings by hydronium has been the subject of theoretical investigation[26]. Earlier gas-phase *ab initio* calculation[26] of the benzene-hydronium potential energy surface finds that a transition state for protonation connects a benzene-hydronium encounter complex to an exit complex for protonated benzene to water. The energy change from separated reactants to separated product is found to be −10.5 kcal/mol, and the transition state for the protonation process is found to be energetically lower than both the reactant and the product, connecting in one direction to an encounter complex of hydronium and benzene, and in the other direction to an exit complex of protonated benzene and water.

**Figure 5:**
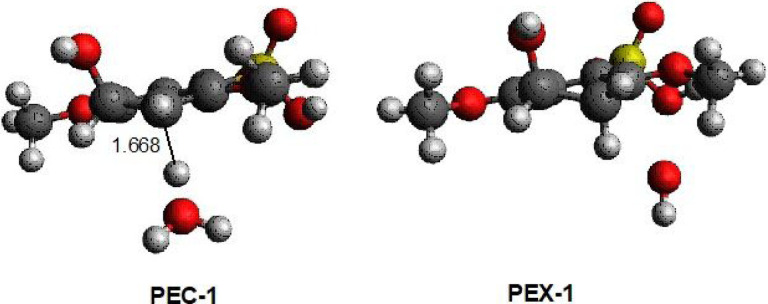
Optimized structures of hydronium-SPEEK1 encounter complex PEC-1 and sPEEK1+-water exit complex PEX-1. The C-H separation in PEC-1 is 1.668 angstroms.

**Figure 6:**
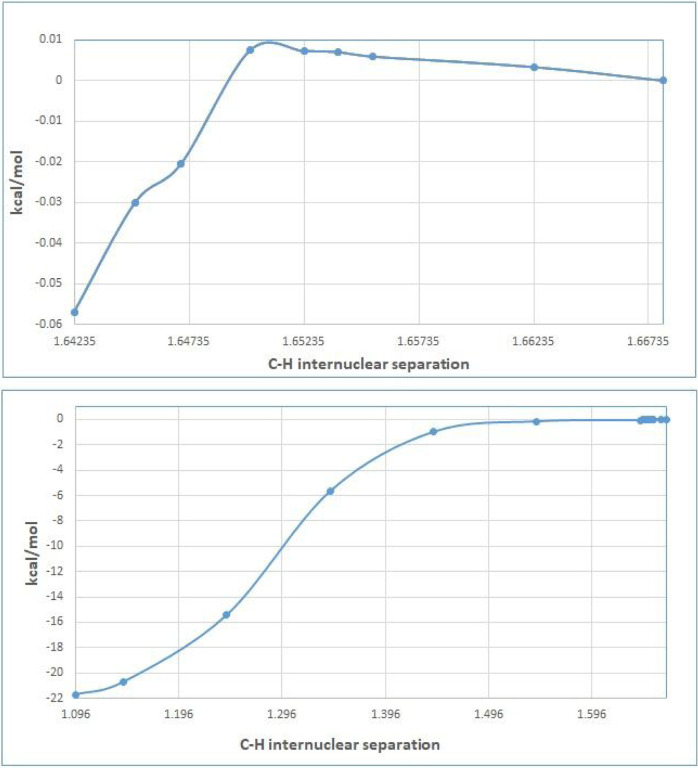
Plot of electronic energy as a function of C-H separation. The top shows the maximum at 1.650 angstroms relative to PEC-1, the bottom plot shows the progression of energies from 1.668 angstrons to 1.096 angstroms (**PEC-1** to **PEX-1**)

M062x/6–311+G(2d,2p) optimizations locate an encounter complex of **SPEEK1OH3** with the hydronium molecule (**H3O**+), **PEC**-**1**. (see Figure 5). A protonation transition state from **PEC**-**1** to a complex of water and **SPEEK1OH3H2**+ is not presented in this work. A series of constrained geometry optimizations[27] in which the C-H internuclear distance shown in Figure 6 is fixed at values between the optimized distance of 1.668 angstrom and values near 1 angstrom. All other nuclear coordinates are permitted to optimize. The resulting energies from these calculations are plotted in Figure 6. Online Resource 2 of the Supplementary Information provides a table displaying the C-H internuclear distances and energies of the resulting structuers relative to PEC-1. Online Resource 2 also provides Z-matrices for the structures resulting from the constrained optimizations.

Figure 6 displays an extremely flat potential energy surface. Of the points plotted, the maximum potential energy relative to PEC-1 is found at a C-H distance of 1.650; the relative energy is only ~0.07 kcal/mol greater than PEC-1. At distances smaller than 1.65, the potential energy decreases to values below the electronic energy of PEC-1. The smallest C-H distance for constrained optimization represented in this figure is 1.14235 angstroms; when this geometry is made a starting point for a full optimization, a complex of **SPEEK1OH3H2**+ and water is optimized. This complex, **PEX-1**, has a C-H internuclear separation of only 1.096 angstroms. The electronic energy is 21.7 kcal/mol lower than that of **PEC-1**.

Figure 6 suggests that little or no energetic barrier exists to protonation of SPEEK1OH3.

#### 3.5.3. Elimination of water

**Figure 7:**
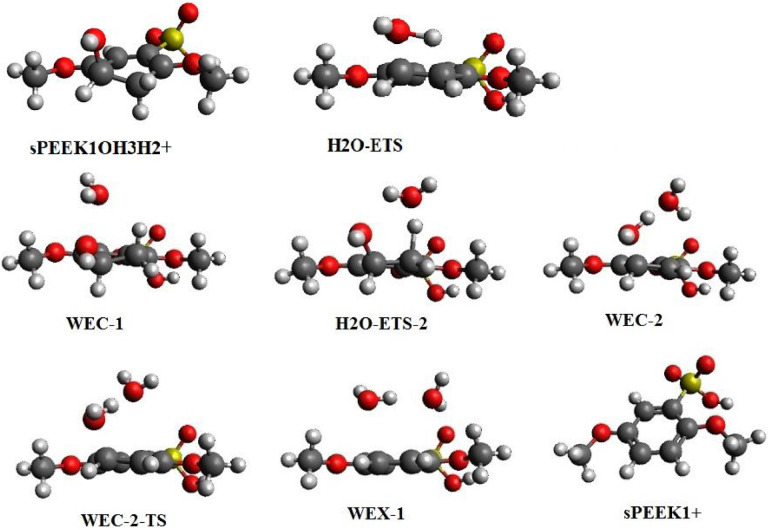
Structures of protonated sPEEK-hydroxy adduct **SPEEK1OH3H2**+, and transition states, intermediates, and product for water elimination reactions.

**SPEEK1OH3H2**+ may eliminate water to form a cationized SPEEK1 molecule (**SPEEK1**+). Elimination occurs through the transition state **H2O-ETS** as displayed in Figure 7. IRC calculations establish the connectivity of this transition state to **SPEEK1OH3H2**+ and to the SPEEK1 cation **SPEEK1**+ and a water molecule in the other. The thermodynamics of the reaction pathway are displayed in Table 8. As seen in Table 8, the barrier to the reaction is extremely high relative to the protonated hydroxyl-sPEEK complex.

Water elimination after protonation may also occur via a second process involving a reaction of **SPEEK1OH3H2**+ with an explicit water molecule; note that this process lies on the potential energy surface of the reactions of sPEEK model molecules with OH radicals and H_3_O^+^.

The resulting reaction may be written

$$\text{SPEEkK}1OH3H2+\;+{\text{H}}_{2}\text{O}\;\;\;à\;\text{SPEEK}1+\;+2{\text{H}}_{2}\text{O}$$

The transition state **H2O-ETS-2** connects to a hydrogen-bonded complex of the reactants, water and **SPK1OH3H2**+, referred to as **WEC-1**. In the other direction, this transition state connects to minimum **WEC-2**, which exhibits one water molecule hydrogen bonded to a second water; the second water interacts with an **SPEEK1**+ molecule via a donative interaction between a lone pair on the water and the half-empty aromatic bonding orbital on the cation. This is a metastable structure with enthalpy and free energy greater than the final products; the final products can be reached by transition state **WEC-2-TS**, which connects **WEC-2** to a hydrogen bonded exit complex of a SPEEK1 cation and two water molecules, referred to as **WEX-1**. The separated products may be reached from **WEX-1** lie at an enthalpy of −8.2 and free energy of −9.1 relative to **SPK1OH3H2+ +** H_2_O. Figure 7 displays optimized structures on the reaction path. The energetics of this reaction relative to **SPEEK1OH3H2+ +** H_2_O are displayed in Table 9.

While hydronium catalyzes this elimination reaction by protonation, the water molecule acts as the co-catalyst for hydrogen atom transfer from a carbon atom to the OH group. The participation of water as a co-catalyze produces a relative barrier of 23.1 kcal/mol vs 39.4 kcal/mol, or a lowering of the barrier by 16.3 kcal/mol. A similar lowering of a hydrogen-transfer barrier appears in a computational study of the transfer of a hydrogen atom from sulfur to oxygen within the thioformic acid molecule in an gas-phase environment[28]; here, a barrier to transfer of 33 kcal/mol is lowered by 21 kcal/mol when a water molecule is included to facilitate the transfer.

## 4. Discussion And Conclusion

The thermodynamic formulation of transition state theory[29,30] expresses rates of reaction in terms of the equilibrium between reactants and transition state, typically presenting rate constants which depend exponentially on the negative of the free energy difference between reactants and transition state. In this formulation, the lower the free energy of a transition state is, relative to the reactants, the faster the reaction might be expected to proceed.

Table 10 displays computed M062X/6–311+G(3df,2p)// M062X/6–311+G(2d,2p) free energies of products and transition states for reactions studied in this work. All energetics in this table are reported relative to the free energy of reactants, which consist of OH, H_3_O+, and one of the model molecules presented in Figure 2. For the acid-catalyzed water elimination reactions, **sPEEK1** is the model molecule; in the case of the chain-breaking reactions at site 1 or 4, results are presented for the corresponding phenyl-substituted species **sPEEK1PH1** or **sPEEK1PH4**, respectively.

This work presents the first density functional computation of the acid-catalyzed water elimination reaction of hydroxyl radicals with the sPEEK polymer. This reaction, like all others computed in this work, proceeds from the addition of an OH radical to a carbon on the aromatic ring of the polymer. Table 10 shows that the reaction is the most exergonic of the reactions studied by at least 4.4 kcal/mol. However, direct elimination of water after protonation at an aromatic carbon adjacent to the hydroxy addition site must proceed through a transition state with a free energy greater than that of the reactants by 7.5 kcal/mol. The participation of a water molecule acting as a co-catalyst to effect the transfer of the H atom from the adjacent carbon creates a much lower barrier to the reaction; the free energy of the transition state with the water co-catalyst is 8.8 kcal/mol lower than the sPEEK1 + OH + H_3_O^+^ reactants.

The main chain of the sPEEK polymer contains ether-bridged aromatic rings; some of which contain sulfonyl groups for the purpose of proton exchange. Chain breaking elimination reactions would be predicted to result from the addition of OH radicals to aromatic carbons bonded to the oxygen atoms of the ether bridge. In the event that no −SO_3_H groups are adjacent to these carbons, the best model for the addition-elimination reaction is provided by the computed reaction pathway for the reaction of OH with **sPEEK1PH4**. While the chain breaking reaction is exergonic, the free energy of the transition state for this reaction is only slightly lower than that of the reactants at −1.7 kcal/mol. If an adjacent −SO_3_H group is present, a chain-breaking reaction may occur with transfer of an H atom from −SO_3_H to the oxygen atom; the best model for that process in this work is the computed reaction of OH with **sPEEK1PH1**; the free energy relative to reactants of the transition state for this reaction, is substantially lower, −10.1 kcal/mol. Addition elimination reactions producing −SO_3_H are modeled by the addition of OH to **sPEEK1** at site 6; this also has a low relative free energy, −11.3 kcal/mol.

The acid-catalyzed water elimination reaction, co-catalyzed by water, has a transition state with a low free energy relative to reactants. Calculations of protonated adducts of **SPEEK1OH3** has produced results suggesting that the exergonicity of protonation of sPEEK-OH adducts adjacent to the site of OH attachment may correspond to negative partial charges on the adjacent carbon atoms. The distribution of partial charges in OH adducts displayed in Table 7 would then suggest that many aromatic carbon sites on the sPEEK polymer, including those with no sulfonyl groups in the aromatic ring (as modeled by **BZ**-**OH**), are likely sites for OH addition followed by protonation at an adjacent carbon atoms with a negative partial charge. The reaction hence might be expected to be a large fraction of the degradation reactions of the sPEEK polymer with OH. Direct addition-elimination chain breaking reactions may only happen at sites where aromatic carbons attach to bridging ether oxygens, and the free energy of the barrier to reaction is greater than that of reactants; this might be expected to be an extremely small fraction of such degradation reactions.

Reactions such as the one reported for **sPEEK1** and **sPEEK1PH1**, in which a hydrogen atom is transferred from a sulfur to an oxygen, or addition elimination reactions producing −SO_3_H, following addition at site 6, have very low free energy barriers, lower than that of the acid-catalyzed, water co-catalyzed elimination reaction, and these reactions might be expected to compete somewhat with a predominant acid-catalyzed, water co-catalyzed elimination reaction.

Additional computational study might further elucidate mechanisms for other degradation reactions. The absence of observation of HSO_3_ product in the experiments described in references 10 and 11 is puzzling in light of the extremely low free energy for the barrier to formation of this product following addition of OH radicals to site 6 reported in this work and in the case of H radicals as discussed in reference 6. One possible explanation may be the presence of a competing reaction that produces oxo-acids of sulfur; these closed-shell sulfur compounds would be undetectable by EPR or ESR methods[10,11] implemented in the experiments described.

In addition for the reaction producing HSO_3_ after addition at site 6, Table 10 includes the possible elimination reactions at site 6 as presented in Table 5. Transition states for these reactions are not reported in this work. However, as seen in Table 5, the reactions producing HSO_4_ and H_2_SO_4_ are endergonic with respect to **SPEEK1OH6**, meaning that free energy barriers to these reactions must be at least as high as the free energies of the products. Therefore, reactions that produce closed-shell H_2_SO4 or open-shell HSO_4_ might be considered uncompetitive as these free energies of products are higher than the free energy barriers reported in Table 10.

H_2_SO_3_ production, however, is highly exergonic with respect to **SPEEK1OH6**. The free energy barrier must be at least equal to the relative free energy of **SPEEK1OH6**, which, as seen in Table 5, is −12.6 kcal/mol relative to the sPEEK + OH + H_3_O^+^ reactants. The products are H_2_SO_3_, which as a closed-shell molecule would not be detected by electron resonance experiments, and a phenoxy radical; phenoxy radicals are observed in the experiments in degradation involving sPEEK discussed in reference 11. Future work will investigate the transition state to H_2_SO_3_ elimination reactions.

The solvated B3LYP calculations discussed in reference 7 find endergonic H-abstraction reactions for the reaction of OH with sPEEK1. Specifically, free energies of H abstraction from site 2 of sPEEK by OH to form water and sPEEK1 radicals are found to have values of approximately −25 or −39 kcal/mol, with the more exergonic value occurring in the case of deprotonated sPEEK. Future work will confirm the exergonicity of these reactions in neutral and anionic species, as well as determining transition states and barrier heights for the H-abstraction reaction.

## Figures and Tables

**Table 1.** Enthalpies (H) and Free Energies (G) of OH adduct radicals.

Site on SPEEK1	OH adduct	H	G
1	**SPEEK1OH1**	−23.2,−*23.9*	−13.2,−*13.9*
2	**SPEEK1OH2**	−18.3,−*19.1* (* −13.7)	−8.3,−*9.1* (*−4.4)
3	**SPEEK1OH3**	−19.2,−*20.2* (*−19.0)	−9.0,−*10.0* (*−7.8)
4	**SPEEK1OH4**	−20.0,−*20.6*	−10.0,−*10.6*
5	**SPEEK1OH5**	−21.9,−*23.0* (*−16.0)	−11.8,−*13.0* (*−6.1)
6	**SPEEK1OH6**	−21.4,−*21.5*	−12.4,−*12.6*

Values are relative to **SPEEK1** + OH and are in kcal/mol. M062X/6–311+G(2d,2p) values are followed by M062X/6–311+G(3df,2p)//M062x/6–311+G(2d,2p) values, in italic. Values with asterisks (*) are solvated B3LYP/6–311+G(d) values for the addition reaction, in aqueous solvent, from reference 7, for comparison.

**Table 2.** Enthalpies (H) and free energies of formation (G) of adducts of OH with **sPEEK1** and **sPEEK1PH1** model molecules resulting from addition at site 1, followed by enthalpies and free energies of optimized structures of the addition-elimination reaction relative to the sPEEK-OH adduct..

Reactant with OH	H of Formation	G of formation	Relative H of transition state	Relative G of transition state	Relative H of intermediate	Relative G of intermediate	Relative H of product	Relative G of product
**SPEEK1**	−23.2,−*23.9*	−13.2,−*13.9*	5.3,4.7	6.3,5.6	−9.0,−9.6	−10.5,−11.0	−2.9,−3.4	−15.7,−15.1
**SPEEK1PH1**	−24.7,−25.4	−14.2,−14.9	3.8,3.7	4.9,4.7	−15.5,−16.3	−17.0,−17.9	−7.1,−7.7	−20.1,−20.7

M062X/6–311+G(2d,2p) values are followed by M062X/6–311+G(3df,2p)//M062x/6–311+G(2d,2p) values, in italic. Values are in kcal/mol.

**Table 3.** Enthalpies (H) and free energies of formation (G) of adducts of OH with **sPEEK1** and **sPEEK1PH4** model molecules resulting from addition at site 1, followed by enthalpies and free energies of optimized structures of the addition-elimination reaction relative to the sPEEK-OH adduct.

Reactant with OH	H of Formation	G of formation	Relative H of transition state	Relative G of transition state	Relative H of intermediate	Relative G of intermediate	Relative H of product	Relative G of product
**SPEEK1**	−23.2,−23.4	−13.1,−13.9	19.0,19.3	18.6,18.9	4.6,5.0	2.6,3.0	9.1,9.7	−2.6,−2.1
**SPEEK1PH1**	−21.6, −22.3	−10.6 −11.3	9.3,9.5	9.3,9.6	−16.5,−16.6	−17.0,−17.2	−10.1,−9.6	−22.0,−21.6

M062X/6–311+G(2d,2p) values are followed by M062X/6–311+G(3df,2p)//M062x/6–311+G(2d,2p) values, in italic. Values are in kcal/mol.

**Table 4.** Enthalpies (H) and free energies of formation (G) of the adduct of OH with the **sPEEK1** model molecule resulting from addition at site 6, followed by enthalpies and free energies of optimized structures of the addition-elimination reaction relative to the sPEEK-OH adduct.

Reactant with OH	H of Formation	G of formation	Relative H of transition state*	Relative G of transition state	Relative H of intermediate	Relative G of intermediate	Relative H of product	Relative G of product
**SPEEK1**	−21.3,−21.5	−12.4,−12.6	−0.1,0.6	0.6,1.3	−11.3,−10.5	−12.5–11.7	−3.6,−2.6	−15.7,−14.7

M062X/6–311+G(2d,2p) values are followed by M062X/6–311+G(3df,2p)//M062x/6–311+G(2d,2p) values, in italic. Values are in kcal/mol.

* The electronic energy of transition state **SPEEK1OH6TS** is only 0.14 kcal/mol greater than the energy of **SPEEK1OH6**

**Table 5.** DH and DG of elimination reactions of SPEEK1OH6.

Products of Elimination Reaction of sPEEK1OH6	DH of reaction	DG of reaction
**sPEEK1OH6-P2 + H2SO4**	23.3,*18.8*	11.5,*7.1*
**sPEEK1OH6-P3 + HSO4**	25.5,*21.6*	13.8,*9.9*
**sPEEK1OH6-P4 + H2SO3**	−11.5,−*8.2*	−24.7,−*21.4*

M062X/6–311+G(2d,2p) values in kcal/mol are followed by M062X/6–311+G(3df,2p)//M062x/6–311+G(2d,2p) values in kcal/mol, in italic. Values are relative to **SPEEK1OH6**.

**Table 6.** Thermodynamics of protonation reactions of **SPEEK1OH3**.

Reaction	DH	DG
**SPEEK1OH3 +** H_3_O+ -> **SPEEK1OH3H2+ +** H_2_O	−22.2, −*22.0*	−22.0,−21.9
**SPEEK1OH3 +** H_3_O+ -> **SPEEK1OH3H4+ +** H_2_O	0.8,*1.1*	0.8,*1.2*

M062X/6–311+G(2d,2p) values are followed by M062X/6–311+G(3df,2p)//M062x/6–311+G(2d,2p) values, in italic. Values are in kcal/mol

**Table 7.** Mulliken charges on carbon atoms adjacent to site of OH attachment in OH adduct molecules.

OH adduct	Adjacent site with least partial charge, followed by Mulliken charge	Adjacent site with greatest partial positive charge, followed by Mulliken charge
SPEEK1OH1	C6: −0.295895	C2: +0.311261
SPEEK1OH2	C1: +0.213092	C3: +0.229474
SPEEK1OH3	C2: −0.164503	C4: +0.078565
SPEEK1OH4	C3: −0.207607	C5: +0.011635
SPEEK1OH5	C6: −0.523895	C4: +0.008081
SPEEK1OH6	C1: −0.249535	C5: −0.241307
BZ-OH	C2: −0.101056	C6: −0.101056

**Table 8.** Thermodynamics of elimination reaction of **SPEEK1OH3H2**+.

Optimized structure(s)	Relative H	Relative G
**SPK1OH3H2+**	0,0	0,0
**H2O-ETS**	38.7,*38.6*	39.5,39.4
**SPEEK1+ + H2O**	−8.7,−8.2	−19.5,−19.0

M062X/6–311+G(2d,2p) values relative to **SPEEK1OH3H2**+ are followed by M062X/6–311+G(3df,2p)//M062x/6–311+G(2d,2p) values, in italic. Values are in kcal/mol.

**Table 9.** Thermodynamics of elimination reaction pathway for elimination water from **SPK1OH3H2**+ with water co-catalyst.

Optimized structure(s)	Relative H	Relative G
**SPEEK1OH3H2+ + H2O**	0,0	0,0
**WEC-1**	−2.2,−*2.1*	6.1,6.2
**H2O-ETS-2**	12.0,*12.2*	22.9,*23.1*
**WEC-2**	2.1,*2.1*	12.2,12.2
**WEC-2-TS**	2.0,2.1*	12.2,12.3
**WEX-1**	−18.6,−*18.3*	−12.0,−11.6
**SPEEK1+ + 2H2O**	−8.7,−*8.2*	−19.5,−19.0

M062X/6–311+G(2d,2p) values relative to **SPK1OH3H2+ + H2O** are followed by M062X/6–311+G(3df,2p)//M062x/6–311+G(2d,2p) values, in italic. Values are in kcal/mol

* The electronic energy of **WEC-2-TS** is only 0.33 (0.39) kcal/mol greater than **WEC-2**

**Table 10.** Free energy of transition states and products relative to sPEEK model molecule +H_3_O^+^ + OH

sPEEK model	Transition State	G (kcal/mol)	Product	G(kcal/mol
**sPEEK1PH1**	**SPEEK1PH1OH1TS**	−10.1	**PHENOL + SPEEK1PH1OH1-P**	−35.6
**sPEEK1PH4**	**SPEEK1PH4OH4TS**	−1.7	**SPEEK1OH4-P + OCH3**	−32.7
**sPEEK1**	**SPEEK1OH6TS**	−11.3	**SPEEK1OH6-P + HSO3**	−27.2
**sPEEK1**	**H2O-ETS**	7.5	**sPEEK1+ + 2H2O**	−40.8
**sPEEK1**	**H2O-ETS-2**	−8.8	**sPEEK1+ + 2H2O**	−40.8
**sPEEK1**	**N/A**	**N/A**	**sPEEK1OH6-P2 + H2SO4**	−5.5
**sPEEK1**	**N/A**	**N/A**	**sPEEK1OH6-P3 + HSO4**	−2.7
**sPEEK1**	**N/A**	**N/A**	**sPEEK1OH6-P4 + H2SO3**	−33.9
